# Prevalence and risk factors of zoonotic diseases on the human-wildlife-livestock interface in Eastern Cape and KwaZulu-Natal Provinces, South Africa

**DOI:** 10.3389/fvets.2026.1673224

**Published:** 2026-03-30

**Authors:** Noluthando Ngcobo, Lelethu Mdoda, Aart-Jan Verschoor, Ntuthuko Raphael Mkhize, Siphesihle Qange

**Affiliations:** 1 Discipline of Agricultural Management, University of KwaZulu-Natal, Pietermaritzburg, South Africa; 2 Economic Analysis Unit, Agricultural Research Council-Central Office, Pretoria, South Africa; 3 Discipline of Animal Science, University of KwaZulu-Natal, Pietermaritzburg, South Africa

**Keywords:** disease prevalence, human-wildlife-livestock interface, one health approach, risk factors, smallholder farmers, South Africa, veterinary epidemiology, zoonotic diseases

## Abstract

Zoonotic diseases pose a significant threat to food security, rural livelihoods, and public health in Southern Africa, where frequent interactions among humans, livestock, and wildlife facilitate disease spillover. This study investigated the prevalence and risk factors of zoonotic diseases among smallholder livestock farmers in the Eastern Cape and KwaZulu-Natal provinces of South Africa. Primary data were collected from 155 farmers using a structured questionnaire, and the analysis combined descriptive statistics with a multivariate probit model. The findings revealed notable vulnerabilities. Only 2% of farmers had ever tested their livestock for zoonotic diseases, while 9% reported livestock abortions, a potential indicator of reproductive zoonoses. Risky consumption behaviors were common: 32% of farmers consumed raw milk, 17% raw meat, and 9% raw eggs. Reliance on unsafe water sources was widespread, with 60% depending on rivers and 6% on dams for livestock water. Awareness of zoonotic diseases was very limited, as 44% of respondents could not identify any associated risk factors, and 24% cited restricted access to information. Poor biosecurity practices were reported by 22% of farmers, while 16% had direct contact with wildlife and 19% consumed wildlife products, further elevating exposure risks. The multivariate probit model identified significant predictors of zoonotic risk, including goat ownership, hunting and trapping, raw animal-product consumption, age, gender, and education. Strengthening veterinary surveillance, expanding farmer awareness programmes, and investing in safe water infrastructure are critical to reducing zoonotic transmission at the human–wildlife–livestock interface.

## Introduction

Globally, over 60% of emerging infectious diseases are zoonotic in nature, with the majority linked to wildlife reservoirs as reported by Rahman et al. (1). The increased pace of land-use change, population growth, and agricultural expansion has created more opportunities for close interaction between humans, domestic animals, and wildlife, raising the likelihood of pathogen spillover (2). In addition to their health impacts, zoonotic diseases threaten food security, disrupt rural livelihoods, and undermine sustainable development especially in regions where people rely heavily on livestock and natural ecosystems for survival (3).

African, the threat of zoonotic disease is particularly acute because of the continent’s great biodiversity, widespread reliance on small-scale agriculture, and persistent gaps in veterinary and public health infrastructure (4). Rural populations often depend on informal livestock production, bushmeat harvesting, and shared natural resources such as water and grazing land (5). These practices, though essential for livelihoods, heighten the risk of cross-species disease transmission. Furthermore, many zoonoses remain under-reported or poorly understood due to limited diagnostic capacity, cultural practices that shape perceptions of illness, and the fragmented nature of surveillance systems (6, 7). The absence of coordinated, multi-sectoral responses to zoonotic threats has further complicated efforts to predict, detect, and respond to outbreaks in both humans and animals.

Within Sub-Saharan Africa (SSA), the human-wildlife-livestock interface has been acknowledged as a critical zone for the emergence of zoonotic disease. This interface refers to the physical or behavioral space where humans, domestic animals, and wild species interact directly or indirectly, often due to overlapping use of landscapes and resources (8). Population pressure, agricultural expansion, deforestation, and infrastructure development are eroding boundaries between settlements, grazing land, and conservation areas (9). In many rural areas, livestock graze on communal lands adjacent to protected wildlife reserves, and people collect firewood or water from forests and rivers that are also frequented by wild animals (10). These shared spaces facilitate the spread of pathogens across species and increase the risk of local outbreaks.

South Africa is a middle-income country with both advanced and under-resourced sectors (11), it sits at the intersection of modern public health systems and traditional, subsistence-based agricultural practices. While urban areas have access to formal veterinary and healthcare services, many rural provinces remain underserved. In provinces such as the EC and KZN, smallholder livestock farming continues to play a pivotal role in local economies and cultural life. Farmers in these areas often operate within communal systems where herds mix freely, biosecurity measures are limited, and proximity to wildlife habitats is common (12, 13). These conditions mirror those observed in other high-risk settings across SSA but are intensified by South Africa’s unique socio-political and environmental landscape.

The EC and KZN are particularly important to consider due to their high levels of livestock ownership, large rural populations, and close proximity to wildlife-rich areas. In the EC, communal livestock farming is the dominant production system, with cattle, goats, and sheep moving across vast tracts of unfenced land (14). Livestock serve not only as sources of income but also play critical roles in social and cultural practices (15). However, the mixing of herds, poor access to veterinary services, and limited awareness of zoonotic risks create ideal conditions for the spread of animal-borne diseases (16). KZN shares many of these characteristics but also contains several protected areas, including Hluhluwe-iMfolozi Park and the iSimangaliso Wetland Park. In many northern parts of the province, communities live in close proximity to these reserves, and human-wildlife interactions whether through hunting, water collection, or conflict are common. The humid climate and seasonal rainfall patterns in the province further support the survival and spread of vector-borne and waterborne zoonoses (17).

The emergence of zoonotic diseases at the human-wildlife-livestock interface is increasingly recognized as a growing threat to rural livelihoods and public health in South Africa. However, in provinces such as the EC and KZN, there is limited empirical data on the prevalence of these diseases and the local risk factors that facilitate their transmission. Without a detailed understanding of how SHFs’ interact with livestock and wildlife and how these connections are influenced by socio-economic, cultural, and environmental conditions it is difficult to design effective, locally appropriate disease prevention and control strategies. There is therefore an urgent need for research that moves beyond disease-specific surveillance to a broader investigation of the interface dynamics, behavioral practices, and structural vulnerabilities that shape zoonotic risks in these high-risk settings.

This study seeks to investigate the prevalence and risk factors of zoonotic diseases at the human-wildlife-livestock interface in selected rural communities in the EC and KZN provinces. By identifying key socio-demographic, behavioral, and environmental drivers of exposure, the study aims to inform the development of integrated, context-specific strategies to reduce zoonotic transmission in smallholder farming systems.

### Theoretical framework

This study applies the One Health approach to examine zoonotic disease risks at the human–wildlife–livestock interface in South Africa. One Health emphasizes the interconnectedness of human, animal, and environmental health (18, 19). While the concept is widely recognized globally, its relevance here lies in rural smallholder farming systems where frequent and often unregulated contact among people, livestock, and wildlife creates opportunities for zoonotic spillover (12).

In the Eastern Cape and KwaZulu-Natal provinces, smallholder farmers commonly graze livestock on communal lands bordering conservation areas and depend on rivers or dams also used by wild animals. These overlapping resource uses increase the risk of pathogen transmission across species (13). The One Health framework provides a lens to analyse how human practices such as raw milk consumption or informal slaughter (20), animal health gaps such as limited testing or uncontrolled livestock movement (21), and environmental exposures such as reliance on shared water sources (17) interact to shape zoonotic risks.

The framework also guides the study’s analytical approach. By employing a multivariate probit model, the research accounts for multiple interlinked risk factors influencing exposure simultaneously, rather than treating them in isolation (22, 23). This integrated perspective allows the study to move beyond descriptive reporting and instead identify systemic pathways of zoonotic transmission. In doing so, the One Health approach supports the development of practical, context-specific interventions that bridge veterinary care, public health, and environmental management in smallholder farming systems (3).

## Methodology

### Description of the study

The study was conducted in the Eastern Cape (EC) and KwaZulu-Natal (KZN) provinces of South Africa, both of which have high concentrations of smallholder livestock farmers. These provinces are characterized by extensive rural populations where livestock farming is an integral livelihood activity. Cattle, goats, sheep, and poultry are commonly reared, providing food, income, and socio-cultural value. The EC is marked by communal farming systems and high poverty levels, while KZN combines coastal and inland agro-ecological zones where households often live in close proximity to conservation areas. These conditions make both provinces critical sites for examining zoonotic disease risks at the human–wildlife–livestock interface.

### Research design, sampling procedure, and sample size

This study adopted a cross-sectional research design, which is well-suited for assessing the knowledge and practices of smallholder livestock farmers regarding zoonotic diseases at a specific point in time. A cross-sectional approach was used to collect data from the study population at a single point in time, providing a snapshot of current conditions and behaviors. A multi-stratified sampling method was employed to ensure representative coverage across the study area’s diverse demographics and livestock practices (33, 34). The minimum sample size (*n* = 150) was estimated using Cochran’s formula for proportions, which provides a baseline for large or undefined populations. However, to ensure representativeness across diverse ecological and demographic contexts, a three-stage multi-stratified sampling procedure was applied. In stage one, districts were purposively selected based on livestock density and proximity to wildlife. In stage two, municipalities and villages were randomly chosen, and in stage three, farmers were selected using systematic random sampling. To minimize potential sampling error introduced by this approach, the sample size was slightly increased to 155 respondents to account for possible non-responses, thereby maintaining statistical reliability. The formula is as follows:


$$n_{1}=\frac{z2p(1-p)}{e^{2}}$$

Where;

*n* = required sample size.

Z = confidence level at 95% (standard value of 1.96).

p = estimate of smallholder livestock farmers which is at 0.89. This was an assumption that 89% of households participates in livestock production in the study area.

q = this is the weighting variable given by 1- p.

$e^{2}$ = margin of error at 5% (standard value of 0.05).

$n_{0}$ = $\frac{\left(1.96)2(0.11)(0.89\right)}{(0.05)2}$ =150.4.

Based on this calculation, the minimum required sample size was 150. However, to account for potential non-responses or incomplete questionnaires, the sample size was increased to 155 to ensure data reliability and representativeness.

### Data collection

The study made use of primary data, collected using a structured questionnaire developed in consultation with animal health experts and administered by trained enumerators. To ensure reliability and validity, the questionnaire was pretested to identify unclear or potentially biased questions, allowing for necessary refinements. This was done using a 10% interval. The pre-testing similarly assisted in training the enumerators and enhancing their understanding of the questionnaire. The Face-to-face interviews were conducted in IsiXhosa and IsiZulu to minimize language barriers, allow farmers to express themselves, and improve response quality. The questionnaire comprised five sections: (i) farmer demographics, natural resources, livestock keeping activities and livestock management activities; (ii) knowledge and practices of livestock farmers towards zoonotic diseases; (iii) the prevalence of the disease along with potential risk factors mainly in the human-wildlife-livestock interface (iv) the vulnerability and adaptive response of smallholder livestock farmers to zoonotic diseases, (v) last section had questions on effects of the disease on livestock farmers productivity. Data was collected between December 2024 and January 2025. Ethical approval was granted by the University of KwaZulu-Natal Research Ethics Committee (HSSREC/00007710/2024).

### Data analysis

The collected data were coded and captured in Microsoft Excel before being exported to STATA 17 for comprehensive statistical analysis. Both quantitative and qualitative data were analysed using appropriate techniques, including descriptive statistics and content analysis. To assess the prevalence and risk factors of zoonotic diseases at the human–wildlife–livestock interface in the EC and KZN provinces, the study employed descriptive statistics to summarize key variables and the multivariate probit model to identify and evaluate multiple, potentially interrelated factors influencing disease occurrence.

### The empirical model

, The study will adopt and use a multivariate probit (MVP) model to estimate the risk factors of zoonotic diseases on the human-wildlife-livestock interface. The multivariate probit model is a statistical technique used to analyse multiple dependent binary outcomes simultaneously (35). Unlike univariate probit models that focus on a single binary outcome, the multivariate probit model allows for the joint analysis of several binary outcomes within a single coherent framework.

In this study a multivariate probit model could be used to simultaneously analyze the effects of various risk factors (such as proximity to wildlife, livestock ownership, etc.) on the presence or absence of multiple zoonotic diseases. The multivariate probit is a standard model for modelling correlated binary data, due to its flexibility it has advantages in handling interpretability of parameters and correlation structures (23). The model involves binary dependent variables, where each variable can take on values of 0 or 1 (22). In addition, Chib and Greenberg (22), indicated that model accounts for the correlation between the dependent variables, allowing for the possibility that the outcomes of these variables are related to each other. This enables a more comprehensive analysis of the interdependencies among multiple binary outcomes (22).

, This approach improves efficiency, captures dependencies between outcomes, enhances prediction accuracy, and reduces the risk of Type I errors (36, 37). With applications spanning economics, psychology, and epidemiology, where interdependencies among binary outcomes are prevalent, the multivariate probit model offers a robust analytical framework, overcoming the limitations of separate models and providing comprehensive insights into complex variable relationships.

, In the MVP model, the choices are not mutually exclusive; therefore, farmers can select more than one option simultaneously. To account for prevalence of zoonotic diseases, the MVP model was used to show the interdependence among dependent variables (38). The MVP model can be represented as follows:


$$y_{\mathrm{im}}=\beta_{\mathrm{im}}X_{\mathrm{im}}+\varepsilon_{\mathrm{im},\;}m=1,\ldots \ldots ..,M\;(i=\mathrm{are}\;\text{risk factors})$$ (1)

Through the transformation of unobserved preference in the preceding (Equation 1) into the observed binary outcome the formula for each risk factor option, the study will obtain the following, Equation 2:


$$y_{\mathrm{im}}=1\;\text{if}\;y_{\mathrm{im}}>0\;\text{and}\;0\;\text{otherwise}\;i=\text{risk factors}$$ (2)

$\varepsilon_{\mathrm{im},}$ *m = 1…, M* are error terms distributed as multivariate normal, each with a mean of zero, and variance–covariance matrix V, where V has values of 1 on the leading diagonal and correlations $p_{\mathrm{jk}}$ = $p_{\mathrm{jk}}$ as off-diagonal elements.

The $y_{\mathrm{im}}$ might represent outcomes for *M* different choices at the same point in time, for example, whether an individual owns each of *M* different consumer durables. Alternatively, the $y_{\mathrm{im}}$ might represent *M* outcomes on the same choice at *M* different points in time.

### Data

Table 1 illustrates data that were collected from smallholder livestock farmers.

**Table 1:** Data collected from smallholder livestock farmers.

Variable	Description	Category	Signs
Dependent variable			
Direct contact with infected livestock or animal products	Yes = 1, No = 0	Dummy	-
Consumption-related zoonotic exposure	Yes = 1, No = 0	Dummy	-
Poor biosecurity and husbandry practices	Yes = 1, No = 0	Dummy	-
Independent variables			
Age	Actual years	Continuous	+/−
Gender	Female = 0, Male-1	Dummy	+/−
Level of education	Education levels	Categorical	+/−
Farm experience	Actual years	Continuous	+/−
River	Yes = 1, No = 0	Dummy	+/−
Dam	Yes = 1, No = 0	Dummy	+/−
Cattle	Yes = 1, No = 0	Continuous	+/−
Sheep	Yes = 1, No = 0	Continuous	+/−
Goat	Yes = 1, No = 0	Continuous	+/−
Chicken	Yes = 1, No = 0	Continuous	+/−
Live near wildlife habitat	Yes = 1, No = 0	Dummy	+/−
Direct contact wildlife	Yes = 1, No = 0	Dummy	+/−
Consume wildlife	Yes = 1, No = 0	Dummy	+/−

Source: Field survey, 2025.

## Results and discussion

### Demographic and socioeconomic characteristics of smallholder livestock farmers

This section presents descriptive statistics on the demographic and socioeconomic factors affecting smallholder livestock farmers.

The mean age of respondents is 59 years, indicating a predominantly older farming population. Older individuals may have more farming experience but may also rely more heavily on traditional practices, which could increase exposure risk, especially in close-contact livestock systems. The average land size is 7.16 hectares, indicating small-scale farming operations. Such limited land size can lead to higher livestock numbers, as noted in Table 2, the average livestock units for cattle (12), sheep (12), goats (9), and chickens (12). Higher stocking densities may increase close interactions among animals and between animals and humans, which could potentially facilitate the transmission of pathogens on the farm (39). About 86% of respondents are male, possibly reflecting a male-dominated livestock production system. This means men are likely the ones in direct contact with livestock, thus more exposed to zoonotic pathogens. From Table 2, the household size distribution shows that an average household has 6 members. Larger households may face greater health risks due to higher person-to-person contact and shared animal-human environments, but they may also benefit from more labour resources to implement preventive measures (40). The data indicate that 74% of livestock farmers operate on communal or tribal land, while only 26% own their land. Communal land tenure systems often involve shared grazing areas, which can lead to increased contact among livestock from different households. This communal grazing practice has been linked with higher risks of zoonotic disease transmission because of the mingling of animals and the potential for disease spread across herds (41).

**Table 2:** Demographic characteristics of smallholder farmers.

Variable description	*n* = 155	
	Mean	Standard deviation
Age	59	14.21
Land size	7.16	50.50
Household size	6	3.68
Cattle	12	26.72
Sheep	12	23.01
Goats	9	15.42
Chicken	12	18.13

	Frequency	Percentage
Gender		
Male	133	86%
Female	22	14%
Level of education		
No schooling	11	7%
Primary	61	40%
Secondary	78	50%
Tertiary	5	3%
Land ownership		
Owned	40	26%
Communal/Tribal land	115	74%
Main reasons for livestock keeping		
Household consumption	32	21%
Sale of animals	22	14%
Wealth status	80	52%
Sale of animal by-products	11	7%
Religious/traditional practices	10	6%
Water source		
River	93	60%
Dam	9	6%
Water tank	46	30%
Borehole	2	1%
Tap water	5	3%
Access to credit		
Yes	17	11%
No	138	89%

Source: Field survey, 2025.

The majority (60%) of the farmers relied on using rivers as a source of water for their livestock, and only 1% boreholes. The heavy dependency on natural and potentially untreated water sources is concerning since it exposes livestock to waterborne pathogens. Rivers and dams can potentially get contaminated by several things, including human and animal waste, especially in places with poor sanitation facilities (17). *Salmonella enterica*, *Shigella flexneri*, and *Campylobacter jejuni* were among the enteric pathogens discovered in river waters in rural SA (20), emphasizing the danger of waterborne diseases. There is a notable minimal use of boreholes (1%), since borehole water, when properly constructed and maintained, is generally less prone to contamination. The low implementation proportion may be due to factors such as the high costs of installing and maintaining it.

According to the results presented in Table 2, the main reasons farmers in the study area kept livestock were for wealth status, household consumption, sale of animals, sale of animal by-products, and using livestock for religious or traditional practices. There are several uses for livestock, including ensuring food security (24, 25), cultural and social functions such as ancestral rituals, *lobola* (bridal) payment (26). Only 11% confirmed they had access to credit, whereas the majority of farmers (89%) in the study did not have access to credit (see Table 2). This could be because most farmers do not have title deeds to the land they occupy. These findings are in line with Zhang et al. (27), who indicated that due to a lack of collateral, most SHFs do not have access to credit as they are not accepted by financial institutions.

### Prevalence and impact of livestock diseases among smallholder livestock farmers

**Figure 1:**
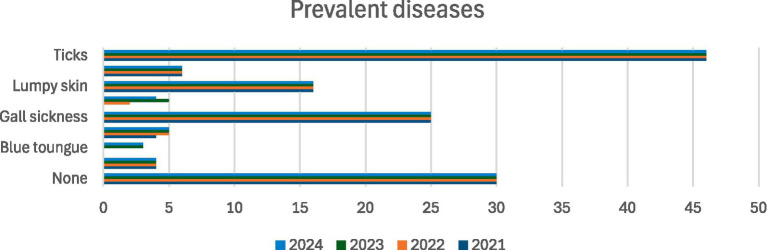
Prevalent diseases over the 2021–2024 period. Source: Field survey, 2025.

Figure 1 presents the prevalence of livestock diseases as reported by smallholder farmers over the 2021–2024 period, revealing a persistent burden of animal health challenges within the sector. The majority of respondents identified ticks and associated tick-borne diseases as the most prevalent health concern across all years. This aligns with prior findings that tick-borne infections continue to dominate within communal and wildlife-adjacent farming systems in South Africa. Among the tick-related illnesses, gall sickness emerged prominently, an infectious condition caused by *Anaplasma marginale*, and commonly transmitted by ticks. Its continued presence reflects inadequate vector control measures and limited access to preventive veterinary interventions. Similarly, lumpy skin disease, a viral illness spread by biting insects and direct animal contact, was consistently reported and poses serious threats to cattle populations, especially in regions like EC and KZN, where seasonal outbreaks are exacerbated by climatic fluctuations and unregulated animal movement (43). While lumpy skin disease and gall sickness are not directly zoonotic, their impact on animal health, productivity, and market access is significant and ultimately affects the economic stability of smallholder farmers.

Other diseases, including sheep scab, heartwater, and diarrhoea, although less frequently reported, were present throughout the four-year period, indicating ongoing endemicity. Heartwater, a tick-borne disease caused by *Ehrlichia ruminantium*, remains a major cause of morbidity and mortality in cattle and goats, particularly in tick-prone zones (42). Diseases like anthrax and blue tongue appeared to have a lower prevalence in the dataset; however, this may reflect underreporting or diagnostic challenges in remote and under-resourced areas rather than a true absence. Interestingly, a consistent subset of approximately 30 farmers per year reported no disease occurrence, which could suggest better animal health management practices, lower exposure risks, or possible gaps in disease recognition.

### Common risk factors associated with zoonotic diseases in the study area

**Figure 2:**
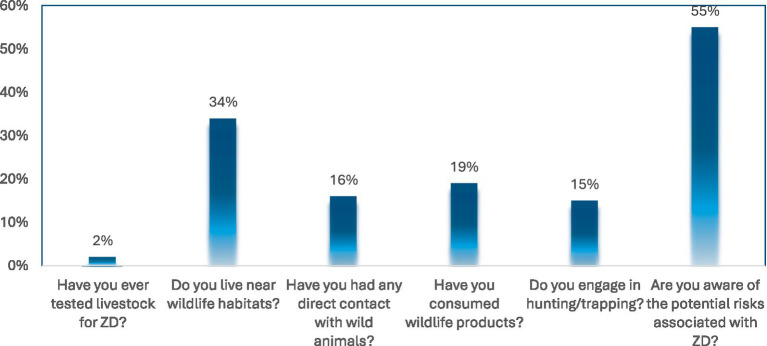
Common risk factors associated with zoonotic diseases. Source: Field survey, 2025.

Figure 2 reveals a complex landscape of zoonotic disease risk in the study area. The most reported factor was awareness of zoonotic risks, with 55% of respondents indicating they were aware of the potential health threats posed by zoonotic diseases. A significant 34% of respondents reported living near wildlife habitats, exposing them and their livestock to direct and indirect interactions with wildlife, an established pathway for zoonotic spillover. Risk is further intensified by behaviors such as consuming wildlife products (19%), direct contact with wild animals (16%), and engaging in hunting or trapping (15%), all of which are associated with elevated zoonotic transmission potential. Only 2% of respondents had ever tested their livestock for zoonotic diseases, highlighting a major deficiency in surveillance and early detection mechanisms.

### Challenges faced by farmers that increase the risk of zoonotic diseases

**Figure 3:**
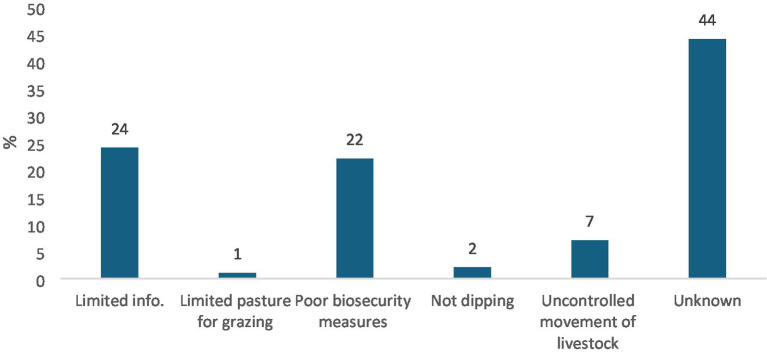
Challenges faced by smallholder livestock farmers. Source: Field survey, 2025.

The data illustrated in Figure 3 reveal multiple interrelated challenges that contribute to farmers’ vulnerability to zoonotic diseases. Foremost among these is the alarmingly high lack of awareness, with 44% of respondents indicating they were not aware of any risk factors associated with zoonotic disease transmission. This substantial knowledge gap implies that many farmers may not recognize the signs of disease, understand transmission pathways, or take appropriate preventative measures, ultimately endangering both animal and human health and undermining broader public health and biosecurity efforts. These findings concur with Munyaneza et al. (21), who highlighted that low awareness and high-risk practices are prevalent amongst smallholder livestock farmers, intensifying zoonotic threats. Furthermore, 24% of respondents cited limited access to information, pointing to systemic failures in communication and extension services that leave farmers ill-equipped to manage or prevent zoonotic risks. This lack of access often results in reliance on informal or traditional knowledge, which may not align with scientifically sound disease prevention strategies. Poor biosecurity was another significant concern, raised by 22% of farmers, reflecting inadequate implementation of basic disease control measures such as isolating sick animals, maintaining sanitation, or properly disposing of waste. These lapses make farming areas especially vulnerable to zoonotic outbreaks, particularly in deep rural areas with limited veterinary oversight. Though reported less frequently, additional factors such as uncontrolled livestock movement (7%), failure to dip animals against parasites (2%), and limited grazing pasture (1%) also contribute to the risk, particularly in communal grazing systems where animal interactions are frequent and unmanaged. Collectively, these risk factors underscore a complex, multi-layered challenge characterized by knowledge deficits, infrastructural limitations, and systemic weaknesses in animal health management that urgently require coordinated, context-specific interventions.

### Occupational exposure of farmers to risk factors associated with zoonotic diseases

Table 3 provides insight into the eating habits and farm-related activities that increase smallholder livestock farmers’ exposure to zoonotic diseases. Among dietary practices, drinking raw milk was the most common, reported by 32% of respondents. This is a deeply rooted practice in many rural communities, often driven by cultural preferences or the lack of access to milk pasteurization or refrigeration. However, raw milk is a well-documented vector for pathogens such as Brucella spp., *Mycobacterium bovis*, and *Listeria monocytogenes*, (44). These pathogens pose serious health risks to both humans and animals, particularly in areas where livestock are not routinely tested for diseases. Additionally, 17% of farmers consumed raw meat, and 9% consumed raw eggs, exposing themselves to pathogens like *Toxoplasma gondii*, *Salmonella*, and *E. coli* (20). Though less prevalent than raw milk consumption, these practices still represent considerable public health concerns, especially in communities with restricted health education access or safe food handling knowledge.

**Table 3:** Exposure of livestock farmers to risk factors associated with zoonotic diseases.

Risk factors	Exposure	
	Frequency (*n* = 155)	Percentage
Eating habits		
Drinking raw milk and soured (Amasi)	49	32%
Eating raw meat	26	17%
Eating raw eggs	14	9%
Farm activities		
Milking	17	11%
Sleeping in an animal shed	0	0%
Dealing with diarrheic animals	7	5%
Assisting a cow during calving	11	7%
History of animal abortion	14	9%
Dispose aborted foetus with naked hands	12	8%
Test animals for zoonotic diseases before purchasing	1	0.65%
Food consumption pattern		
Do you regularly consume raw milk and soured (Amasi) from your animals?	120	77%
Who mainly consumes milk in the household	134	86%
How often is milk consumed? Weekly	103	66%
How do you process meat with abnormal spots or sick animal? Overcook meat	125	80%

Source: Field survey, 2025.

Direct contact with livestock through routine farm activities also contributes to zoonotic disease exposure. While milking was reported by 11% of respondents, it still presents risk if done without protective measures, as milk can carry infectious agents even from asymptomatic animals (28). Assisting animals during calving (7%), handling diarrheic animals (5%), and disposing of aborted foetuses with bare hands (8%) are particularly concerning due to the high potential for contact with infectious fluids and tissues. These practices expose farmers to pathogens such as *Brucella* spp., *Leptospira* spp., and *Cryptosporidium*, which are transmitted through reproductive and gastrointestinal secretions (13). Additionally, 9% of farmers reported a history of animal abortions on their farms a red flag that may indicate the presence of underlying reproductive zoonoses. The lack of personal protective equipment and biohazard disposal protocols further amplifies the health risks posed by these practices, reflecting a pressing need for on-farm training in safe livestock handling.

Most alarmingly, only one respondent (0.65%) reported testing animals for zoonotic diseases before purchase, underscoring an almost complete absence of preventive disease screening in local livestock trade. This is particularly dangerous in communal farming settings, where the introduction of infected animals into herds can lead to rapid and widespread disease transmission. The findings align with Munyaneza et al. (21), who documented similar gaps in livestock health screening across informal markets in Southern Africa. On a more positive note, none of the farmers reported sleeping in animal sheds, a practice which, while common in some high-risk regions, can significantly increase exposure to airborne zoonotic pathogens. Nonetheless, the overall pattern of risky dietary behaviors, unsafe animal handling practices, and lack of disease prevention measures illustrates a systemic vulnerability to zoonotic disease outbreaks, one that demands urgent attention through targeted education, improved veterinary support, and community-based surveillance interventions.

### Assessment of multicollinearity using the variance inflation factor (VIF)

Table 4 shows the Variance Inflation Factor (VIF). Values begin at 1, with 1 indicating no correlation among predictors, values between 1 and 5 suggesting moderate correlation, and values above 5 indicating high multicollinearity (29). The results presented in Table 4 reveal that all predictor variables have VIF values well below the critical threshold of 10, with a mean VIF of 1.36, indicating a very low level of multicollinearity across the model. The highest VIF recorded was 1.73 for the variable consumed wildlife, and the lowest was 1.02 for level of education, further confirming the absence of problematic intercorrelations. Therefore, based on the VIF diagnostic test, the regression model is statistically robust with respect to multicollinearity, ensuring reliable interpretation of the estimated coefficients and the overall performance of the model.

**Table 4:** Diagnostic to assess the degree of multicollinearity.

Variable	VIF	1/VIF
Consumed Wildife	1.73	0.578
Live near wildlife habitat	1.55	0.645
Hunting/Trapping	1.51	0.661
Do you know ZD	1.50	0.665
Aware of potential risks	1.45	0.688
Gender	1.09	0.929
Consumed raw products	1.06	0.946
Level of education	1.02	0.985
Mean VIF	1.36	

Source: Field survey, 2025.

### Multivariate probit regression results estimating risk factors of zoonotic diseases on the human-wildlife-livestock interface

The MVP regression model presented in Table 5 demonstrates a modest overall model fit, with varying degrees of explanatory power across the three dependent variables: Zoonotic Disease Testing, Household Member Affected, and Experience with Zoonotic Disease. All four tests (Wilks’ lambda, Pillai’s trace, Lawley-Hotelling trace, and Roy’s largest root) were highly significant (*p* < 0.001). However, due to the nature of the data (self-assessment scores of risk factor attributes), the study reports the Pillai’s trace statistics (F 2.25) because of its robustness and insensitivity to assumptions on normality. The R-squared values are low, as commonly expected in studies that involve cross-sectional data. The Breusch-Pagan test result of Chi^2^ (3) = 3.800 with a *p*-value of 0.284 suggests there is no statistically significant correlation between the error terms of the equations in the multivariate probit model. A high *p*-value, like 0.284, means there is no strong evidence that the errors in the three equations are connected. The multivariate probit regression model analysed three binary outcomes: (i) Direct Contact with Infected Animals or Animal Products, (ii) Consumption-Related Zoonotic Exposure, and (iii) Poor Biosecurity and Husbandry Practices.

**Table 5:** Multivariate probit regression estimating risk factors of zoonotic diseases on the human-wildlife-livestock interface.

	Direct contact with infected animals or animal products			Consumption-related zoonotic exposure			Poor biosecurity and husbandry practices		
	Coeff	T	*p* > |t|	Coeff_Y_	T	*p* > |t|	Coeff	T	*p* > |t|
Age	0.000	0.19	0.847	0.087	0.13	**0.000** ^***^	0.001	0.31	0.757
Gender	−0.002	−0.14	0.890	0.025	0.53	**0.028** ^**^	−0.112	−1.09	0.279
Level of education	−0.004	−0.50	0.619	0.020	0.75	0.454	0.013	0.22	**0.050** ^**^
Farm experience	−0.000	−1.60	**0.027** ^**^	0.004	0.28	0.783	0.004	1.17	0.244
River	−0.006	−0.60	0.548	−0.004	−0.12	0.906	0.026	0.34	0.735
Dam	0.021	1.08	0.284	0.120	1.62	0.109	−0.122	−0.75	0.455
Cattle	−0.000	−1.91	0.059	0.000	0.25	0.805	−0.001	−0.47	0.641
Sheep	0.001	0.25	0.803	−0.000	−0.22	0.827	−0.000	−0.14	0.891
Goat	0.002	4.23	0.000***	−0.000	−0.32	0.752	0.001	0.35	0.727
Chicken	−0.001	−2.37	0.019**	−0.000	−0.37	0.712	−0.000	−0.20	0.838
Live near wildlife habitat	−0.011	−0.79	0.430	0.033	0.64	0.524	0.001	0.01	0.995
Direct contact wildlife	0.020	1.20	0.232	0.058	0.92	0.357	0.068	0.49	0.625
Consumed wildlife	−0.017	−1.08	0.284	−0.028	−0.47	0.641	0.062	0.46	0.644
Hunting/trapping	0.087	3.65	0.000***	0.120	−1.32	0.027**	−0.068	−0.34	0.733
Bitten scratched	0.018	1.06	0.293	0.130	−2.00	0.048**	0.167	1.16	0.248
Consume raw products	0.034	2.22	0.029**	−0.057	−0.96	0.337	0.082	0.62	0.537
Aware of potential ZDRisk	0.009	0.54	0.592	−0.068	−0.93	0.353	0.337	1.48	0.142
Do you drink raw milk	0.007	0.36	0.717	−0.012	−0.22	0.824	−0.182	−1.13	0.263
Do eat raw meat	−0.021	−1.54	0.126	0.074	1.29	0.198	0.88	0.76	0.449
Do you eat raw eggs	0.002	0.15	0.884	0.153	2.65	0.009***	−0.196	−1.55	0.123
Do you milk	−0.028	−1.84	0.068*	−0.026	−0.62	0.539	0.004	0.03	0.972
Do you deal diarrheic	0.003	0.28	0.782	0.006	0.14	0.887	−0.168	−1.83	0.070***
Do you assis during calving	−0.003	−0.28	0.776	−0.057	−1.22	0.223	0.147	1.60	0.112
History of animal abortion	−0.002	−0.17	0.864	0.028	0.51	0.610	0.143	1.38	0.169
Do you dispose placenta	0.007	0.63	0.531	0.052	1.03	0.304	−0.069	−0.58	0.563
Sanitation situation	0.009	1.12	0.262	−0.016	−0.52	0.607	0.054	0.49	0.628
Waste disposal	0.015	1.24	0.217	0.042	0.91	0.363	−0.170	−1.66	0.020**
Cons	−0.010	−0.34	0.737	−0.052	−0.45	0.654	0.097	0.38	0.705
Joint model		*F* = 2.25***							
Corrected model		*F* = 2.36***			*F* = 2.10***			*F* = 3.68***	
R-squared		0.14			0.11			0.18	
Breusch-pagan test		Chi^2^ (3) = 3.800	Prob > χ2 = 0. 284						

***, **, *, significant at 1%, 5% and 10% significant level, respectively. Source: Field survey, 2025.

From the first dependent variable Direct Contact with Infected Animals or Animal Products, the findings show that goat ownership is significant at the 1% level. This implies there is a positive relationship between owning goats and the increased risk of direct contact-related zoonotic exposure. This may reflect higher vulnerability of goats to zoonoses such as brucellosis or Q fever, which has been noted to prompt greater disease management vigilance. This is in line with Munyaneza et al. (21), who reported heightened awareness and disease management among goat farmers. Conversely, chicken ownership is significant at 1% and has a negative relationship. This could be due to the lower perceived economic value or zoonotic risk associated with poultry, as suggested by Grace et al. (30). Hunting/trapping is a very strong predictor and is highly significant at 1%. This suggests that direct interaction with wildlife through hunting significantly raises exposure risk. Hunting brings humans into close contact with bodily fluids, tissues, and carcasses of wild animals, which are often reservoirs for diseases like tuberculosis, brucellosis, and rabies (31). Farm experience is significant at 5% indicating that longer farm experience is associated with lower risk of direct zoonotic exposure. This suggests that seasoned farmers may be more familiar with safe animal handling or have developed informal risk-mitigation practices through experience (32). Lastly, consumption raw animal products is significant at 5% indicating that consuming raw products like unpasteurized milk or undercooked meat may also involve direct contact (e.g., during preparation), raising exposure risk.

For the second dependent variable consumption-related zoonotic exposure, the results show that hunting/trapping is significant at the 5% level. Being bitten or scratched by wildlife is significant at 5%, indicating a positive relationship between being bitten or scratched by wildlife and the exposure to zoonotic diseases. This is in line with Ramadhan (9), who reported that physical injury from wildlife remains a confirmed zoonotic transmission route and should be monitored closely. Age is significant at 1% and is positively associated with increased risk of consumption-related zoonotic exposure. This implies that older smallholder farmers are more likely to engage in risky consumption behaviors such as eating raw milk, raw eggs, undercooked meat, or consuming products processed without proper hygiene. Furthermore, gender is significant at 5% and has a positive effect on consumption-related zoonotic risk, with males more likely to engage in high-risk dietary practices than females.

The third dependent variable poor biosecurity and husbandry practices waste disposal shows a negative coefficient, significant at the 10% level, suggesting that better waste management may reduce the likelihood of observing zoonotic disease symptoms. Education is significant at 5% and is positively associated with better practices. Suggesting that higher education may correspond to improved husbandry and reduced zoonotic exposure.

## Conclusion and recommendations

The findings of the study revealed critical gaps in awareness, prevention, and surveillance among smallholder livestock farmers. Only 2% of farmers had ever tested their animals for zoonoses, and nearly half (44%) were unaware of any risk factors, underscoring a severe knowledge deficit. Risky practices—such as consuming raw animal products, poor biosecurity, direct contact with wildlife, and hunting—were significant contributors to zoonotic exposure. Socio-demographic factors, including age, gender, education, and farming experience, further shaped these risks. The evidence demonstrates that zoonotic disease risks are driven by a combination of behavioral, environmental, and systemic vulnerabilities. Addressing these challenges requires integrated One Health strategies that bridge veterinary, human health, and environmental interventions.

## Recommendations

- Farmers must adopt safer livestock management practices by isolating sick animals, improving biosecurity, and avoiding the consumption of raw animal products.
- Farmers must actively participate in training and awareness programmes to strengthen knowledge of zoonotic risks and prevention.
- Municipalities must invest in safe water sources, waste management, and sanitation infrastructure to reduce environmental exposure to pathogens.
- Government departments must expand veterinary surveillance, diagnostic testing, and mobile clinic services in underserved rural communities.
- Government departments must strengthen interdepartmental collaboration through a One Health approach that links animal health, human health, and environmental management.

## Data Availability

The datasets used or analyzed during the current study are available from the corresponding author upon reasonable request.
